# Sport and exercise as medicine in the prevention and treatment of depression

**DOI:** 10.3389/fspor.2023.1136314

**Published:** 2023-03-09

**Authors:** Klaus W. Lange, Yukiko Nakamura, Katharina M. Lange

**Affiliations:** ^1^ Faculty of Human Sciences (Psychology, Education and Sport Science), University of Regensburg, Regensburg, Germany; ^2^ Department of Psychology, University of Bath, Bath, United Kingdom

**Keywords:** depression, mental health, sport, exercise, prevention, treatment

## Introduction

Emerging evidence has demonstrated an impressive link between physical activity and improved mood. While evidence of beneficial mental health effects of exercise is mounting, doctors, psychologists and other health practitioners are slow to consider exercise and seldom use it as a therapy tool. The present opinion piece argues that, in view of the unsatisfactory mitigation of disease burden and limited long-term effectiveness of the available treatments for depressive disorders, physical exercise and sport deserve far greater consideration and may become a viable measure in the prevention and treatment of depression. The available research findings make a robust case for exercise as a means of protecting and improving mental health as well as physical health.

Depression is highly prevalent across the entire lifespan worldwide and has become the leading cause of burden of disability globally (1). Depression is highly debilitating, causing reduced quality of life and severe functional disability, and because of its high rate of recurrence, depression is difficult to treat (2). The therapies available for major depressive disorder remain less than satisfactory, with a significant proportion of people treated for depression remaining refractory to treatment. The long-term effectiveness of pharmacotherapy, such as the administration of selective serotonin reuptake inhibitors, and psychological treatment, such as cognitive behavioural therapy, remains a matter of debate, and the capacity of these treatments to ameliorate the cumulative burden of depression on society is limited. There is therefore an urgent need to find novel approaches to the prevention and treatment of depression. The major impact of lifestyle on the development of depression often goes unrecognised, and an ever-growing body of research findings suggest a significant contribution of maladaptive lifestyle choices to the pathogenesis of the disorder. Available evidence supports the view that modifiable lifestyle factors, such as diet and physical exercise, should be considered in the management of common mental disorders. Lifestyle-oriented interventions may be capable of reducing the occurrence and severity of depressive disorders and could become targets of health campaigns at population level. Diet and exercise modification has also been suggested as a treatment strategy in the management of depressive disorders arising during the current coronavirus pandemic (3–6). While various nutrients and food bioactives have been claimed to be effective in alleviating depression, evidence of their clinical efficacy is lacking (7–10).

## Physical exercise and depression

Regular physical exercise is one of the best and most affordable ways of maintaining good health and improving overall well-being. Physically active individuals have been found to benefit from improved levels of health-related fitness, a decrease in overall mortality and reduced risk of developing a wide range of non-communicable conditions, such as obesity, cardiovascular disease, diabetes, stroke and cancer (11). The health benefits of exercise have also been widely acknowledged to encompass mental health. For example, a cross-sectional assessment of more than 1.2 million adults in the United States, who were matched for various sociodemographic and physical health factors, found that people who exercised reported 1.49 (43.2%) fewer days of poor mental health in the past month than people who did not exercise, with all types of exercise and sport being associated with a reduced mental health burden (12). Epidemiological research suggests that insufficient physical activity may increase the risk of developing depressive symptoms, while adequate physical activity based on clinical guidelines is associated with fewer symptoms. On the basis of prospective cohort studies objectively assessing the aerobic fitness of participants, preliminary results have demonstrated that individuals with low and medium cardiorespiratory fitness have an elevated risk of developing depression (13). A further meta-analysis investigating the dose-response relationship between physical activity and incident depression concluded that physical activity had significant mental health benefits, even at levels below public health recommendations (14).

While the association between exercise and depression is well established, causation remains a matter of debate. Disturbances of both mental well-being and physical activity may be influenced by a third variable. Unmeasured factors closely related to physical activity may play a role. In view of the social context of physical activity, the interplay between exercise and social interactions or other lifestyle habits may impact mental health. Reverse causation is also possible, with physical activity being driven by mental health rather than lifestyle. For example, changes in lifestyle could be early signs of depression, and individuals with more symptoms of depression may be less likely to participate in sport. The findings of a prospective cohort study have suggested that the association between exercise and depression may be bidirectional (15). Establishing a causal relationship between exercise and mental well-being and health requires randomised controlled trials demonstrating beneficial effects of exercise interventions on depressed mood.

## Antidepressant efficacy of exercise in randomised controlled trials

The debate surrounding the effects of physical exercise on depression has often been contentious, leading to uncertainty regarding the magnitude of these effects. Meta-analyses of randomised controlled trials have reported moderate to large antidepressant efficacy of exercise. The effect sizes reported may have been influenced by inclusion criteria, heterogeneity of samples and diagnostic criteria as well as publication bias. Furthermore, various studies compared exercise in addition to an established therapy vs. an established therapy alone (16). This approach assumes that the efficacies of treatments can be added and subtracted algebraically. However, this assumption does not take into account that exercise may at least partially overlap and interact with the mechanisms underlying pharmacological and psychological therapies of depression. In a meta-analysis adjusting for publication bias, large antidepressant effects of exercise on depression were found in comparison with non-active control conditions (17). The effect was particularly high for studies including people with major depressive disorder. Larger effect sizes were also found in patients diagnosed with depression but without other clinical co-morbidities. Exercise programmes have also been proposed as a promising means of protecting against the development of depressive symptoms in young people (18). Moreover, findings from randomised controlled trials suggest a moderate positive effect of exercise interventions on the severity of adolescent depression (19). In the context of the high risk of social isolation in older individuals with depression, group exercise appears to be particularly effective (20). Future studies should therefore address exercise as a means of promoting social interaction. The question of whether exercise in combination with a conventional treatment produces better results than exercise or the other treatment alone remains at present a matter of debate owing to the limited data available. Furthermore, the currently established meta-analytic evidence showing antidepressant efficacy of exercise in clinical settings, with relatively low attrition in people with major depressive disorder, needs to be complemented by randomised controlled trials providing ecological (i.e., real life) evidence. These trials should examine the effectiveness of exercise interventions, defined as benefits produced in daily clinical practice, using wide inclusion criteria and functional health outcomes, such as quality of life.

In addition to the compelling evidence derived from randomised controlled trials demonstrating the efficacy of exercise in the treatment of depression, physical activity and exercise appear to be potentially protective factors for incident depression over the entire lifespan. A reduction in depressive symptoms in people with higher physical activity levels has been shown consistently across different cultures and continents (21). Moreover, physical exercise can be used in the acute management of depressive symptoms, with even a single bout of exercise promoting well-being in people with major depressive disorder (21).

A recent systematic review and network meta-analysis of 21 randomised controlled trials (*n* = 2,551) has examined the comparative effectiveness of exercise, antidepressants and their combination for alleviating depressive symptoms in adults with non-severe depression (22). No differences in therapeutic effectiveness were found between the three interventions, with all treatments being more beneficial than controls. The finding that exercise alleviates symptoms of depression to a similar extent as pharmacotherapy supports the adoption of exercise as an alternative treatment approach for the management of non-severe depression in adults. Furthermore, a network meta-analysis was conducted to compare the effectiveness of distinct exercise types, such as aerobic exercise, resistance exercise, mind-body exercise (such as yoga, Tai Chi and dance), stretching and multimodal exercise, which combines at least two types of exercise, on mental health disorders including depression (23). Aerobic exercise, mind-body exercise and multimodal exercise were shown to have significant effects, with moderate-to-large effect sizes, on depressive symptoms when compared with control conditions (23). These findings suggest that various distinct exercise modalities may have clinically meaningful benefits in the treatment of depression and should therefore be considered as complement to medication. Multimodal exercise was found to have the highest probability of being the optimal exercise for improving depressive symptoms. This suggests that combining various exercise types is likely to contribute to greater improvements in depression. In addition, the result of meta-regression indicated that intervention length (4–12 weeks), rather than exercise frequency and session duration, moderated the positive effects of mind-body exercise (23). This confirmed findings of previous meta-analyses, which had shown that exercise length was the only characteristic related to the effect size for depression treatment (24, 25), with intervention periods of 9–12 weeks being associated with the largest reduction. Future studies should examine long-term exercise interventions lasting more than 12 weeks.

## Mechanisms underlying beneficial effects of exercise on mental health

Both physiological and psychological mechanisms through which physical exercise may exert its beneficial effects on mental health have been hypothesised. These mechanisms include changes in the availability and metabolism of brain neurotransmitters and in sleep regulation. Some evidence suggests that exercise could promote adaptations in regard to neurogenesis, brain structure, cognitive processes related to the prefrontal cortex and various markers of inflammation (26). In addition, epigenetics is likely to play a role in the mental health effects of exercise and sport. Physical activity seems to be capable of modulating stress-induced changes in DNA methylation and gene expression, with positive epigenetic influences of exercise counteracting the negative influences of stress (27). However, the paucity of available findings currently precludes the drawing of any firm conclusions regarding underlying neurobiological mechanisms. Psychologically, an improved perception of overall physical health as a result of regular exercise may boost confidence. Calming the mind by modifying emotional action tendencies and interrupting negative cycles of worry and negative thoughts as well as changes in coping self-efficacy have also been proposed as psychological mechanisms. Engaging in sports involving interaction with other people may reduce feelings of isolation and loneliness, improve social confidence and skills and thus promote positive interpersonal relationships and psychosocial development. These are all critical factors in reducing depressive symptoms. Moreover, substituting exercise for unhealthy ways of coping with depressive symptoms and emotional pain, such as alcohol use and overconsumption of food, may help in developing new and healthier coping strategies.

## Strategies to increase physical activity

Changing a sedentary lifestyle and increasing physical activity normally poses a major challenge. Outcomes of introducing a physical exercise regimen may be improved through a combination of various strategies, such as setting realistic goals, starting with short periods of physical activity, improving self-regulation, strengthening non-conscious processes, improving accessibility to exercise facilities and using internet and smartphone apps. Maintaining a physical exercise regimen presents a further problem. Rates of adherence to prescribed exercise regimens have been found to drop significantly within a few months (28). However, they can be enhanced when written prescriptions provide both a detailed exercise plan and suggestions for overcoming anticipated potential problems and barriers. Elevated drop-out rates have been found in groups undertaking high-intensity training compared to low-intensity programmes (29). Given the higher efficacy of high-intensity programmes, exercise regimens need to be individually tailored in order to maximise the beneficial effects of exercise while minimising the drop-out rate.

Virtual reality applications have been widely used in neurological rehabilitation and have also been shown to be capable of providing positive effects for a number of mental health conditions. In particular, virtual reality exposure therapy appears to be a successful tool for the treatment of depression. In this context, several empirical studies investigating potential benefits of virtual reality exercise have found significant improvements in depression-related measures (30). In view of the steady increase in the number of people with depression during the COVID-19 pandemic, web-based exercise interventions, such as those using websites, mobile apps and emails, have also emerged as potential therapeutic approaches, especially in times of lockdowns and social distancing. Preliminary findings suggested some benefits of web-based exercise interventions, such as endurance training or combined endurance and strength training, for depressive symptoms (31). Nevertheless, given the dearth of available literature and the lack of high-quality studies, further research is required before the large-scale implementation of virtual reality or web-based exercise interventions in clinical practice can be recommended for the alleviation of depression.

## Limitations of available evidence

Despite the promising findings of numerous studies assessing the efficacy of sport and exercise in depression, several limitations of the available evidence should be considered. Most systematic reviews found a positive impact of exercise or null results, with only a few reporting a negative influence. However, possible negative mental health outcomes of exercise should be explored more thoroughly. For example, the findings of some studies have suggested that over-scheduling with a high degree in participation in organised activities might lead to stress, particularly among affluent youths (32). Most studies have investigated the effectiveness of exercise in individuals with mild-to-moderate depression. Thus, the available findings cannot be generalised to all people with depression and, in particular, the potential effects of exercise in more severe depression remain unknown. In many trials included in the meta-analyses above, participants were receiving antidepressant pharmacotherapy, with treatment regimens varying considerably. Possible interactions between exercise, medication and symptomatology should be investigated in more detail in future studies. Since previous research was usually restricted to single geographic or sociodemographic areas, the generalisation of results to a wider population was limited. Large population-level research is required to address this issue. In addition, while exercise intensity was described using multiple approaches by some authors, it was not quantified by others. Thus, exercise intensity could not be used as a moderator in meta-analyses. More importantly, evidence-based recommendations regarding the intensity of exercise interventions in the prevention and treatment of depression cannot be made at present.

## Conclusion

Further interventional research on sustainable exercise effects in individuals with depression is needed in order to identify the ideal types and amounts of physical activity that may help prevent and treat depression. It would be important, for example, to ascertain whether certain types of exercise and sports have advantages over others and whether the involvement of a person's favoured sport may produce greater benefits. Furthermore, the potential of exercise to potentiate the effectiveness of cognitive behavioural therapy should be explored. The sponsoring of high-quality studies investigating these questions will pose challenges, since immediate pecuniary returns from such investigations cannot be expected. However, long-term gains may include an amelioration of the burden of mental illness and additional physical health benefits in respect of cardiovascular and metabolic diseases. Thus, political interventions, public funding and support through non-profit organisations will be needed to help counter the challenges faced in the fostering and maintenance of public mental health. In addition to specialist care, task-shifting to trained health workers may be capable of providing cost-effective and sustainable global mental health care (33).

In conclusion, a sedentary lifestyle and lack of physical activity are major factors involved in depression. Physical activity and sport, with their global accessibility, significant and clinically meaningful efficacy as well as virtual absence of adverse effects, offer a promising, evidence-based option for the prevention and treatment of depression. Any increase in physical activity should therefore be encouraged by health practitioners in order to improve mental health. Since exercise is a safe and inexpensive approach to the management of depressive disorders, interventions targeting its adoption and maintenance merit far greater consideration in clinical practice and may become an appealing option for public health promotion and sustainable mental health care systems worldwide.

The bottom line is that the mental health of all may benefit from an active lifestyle including the playing of sports. Exercise as medicine should be routinely provided to all individuals with depressive symptoms and should also be included in therapy guidelines for depression.
